# Factors associated with a positive depression screen after a miscarriage

**DOI:** 10.1186/s12888-018-1991-5

**Published:** 2019-01-07

**Authors:** Steve Kyende Mutiso, Alfred Murage, Abraham Mukaindo Mwaniki

**Affiliations:** Department of Obstetrics and Gynaecology, Aga Khan University hospital, Nairobi, Kenya

**Keywords:** Depression, Miscarriage, Factors influencing

## Abstract

**Introduction:**

Miscarriages are a common pregnancy complication and positive depression screen after a miscarriage has been shown to be high in our population. Various factors are associated with an increased risk of developing depression after a miscarriage. However, these factors vary across populations studied with no studies existing in our region. We set out to determine the factors associated with a positive depression screen among post-miscarriage women at the Aga Khan University hospital, Nairobi.

**Methods:**

Patients were recruited at the 2 weeks clinic review after a miscarriage in the gynaecological clinics. They were screened using the Edinburgh postnatal depression scale for depression after a miscarriage. Analysis was done using Univariate and multivariate analysis to compare clinical variables between the screen - positive and screen - negative women in order to delineate the potential pattern of association between the two among the study subjects.

**Results:**

Positive depression screen was detected in 34.1% of the patients recruited. Univariate analysis revealed that education level (*p* = 0.039) and mode of conception (*p* = 0.005) impacted on the outcome of the depression screen. In multivariate analysis, multiple factors impacted on the depression screen and these included: age (*p* = 0.009), education level (*p* = 0.001), gestation at miscarriage (*p* = 0.04), marital status (*p* = 0.043), prior miscarriage (*p* = 0.011) and mode of conception (*p* = 0.03).

**Conclusion:**

Factors that seem to impact on the positive depression screen include a younger age, low education level, an older gestational age at miscarriage, being single, an assisted mode of conception and prior miscarriage. These factors may be used to triage women after a miscarriage in order to pick up those who may screen positive for depression after a miscarriage.

## Background

Miscarriages occur in about 10 to 15% of pregnancies that are considered low-risk [1, 2]. Psychiatric morbidity including depression, anxiety and even post-traumatic stress disorders have been shown to be a complication of miscarriages [3–5]. The prevalence of depression after a miscarriage seems to be the most common of these with rates of 10–20% being reported in literature [6, 7]. Furthermore, in the first publication of this series, we described a high positive screen for depression after miscarriage in our population of 34.1% [8].

Many factors have been associated with a positive depression screen after a miscarriage. Women who have had infertility, history of a depressive disorder and those who were younger in age have been found to be more vulnerable to depression after experiencing a miscarriage [6]. Moreover, those that have had a previous case of pregnancy loss and a prior history of depression have also been shown to have a higher incidence of depression after a miscarriage [7, 9–11]. The type of miscarriage, that is, elective pregnancy termination versus involuntary pregnancy loss, has also been shown to affect occurrence of depression after a miscarriage with depression being higher in the former than the latter [12]. These factors seem to vary according to the population sampled and hence analysis of these factors in our population may aid in identifying women at risk of depression after a miscarriage. It is also important to see whether the factors that impact on occurrence of depression in this population are the same as in other populations hence adding to the worldwide body of literature in depressive illness after a miscarriage.

No studies exist that look at the factors impacting on the occurrence of positive depression screen after a miscarriage in our population hence we set out to determine factors that impact on the occurrence of a positive depression screen after a miscarriage in our population at a private tertiary teaching hospital in Kenya.

## Methods

### Objective

To determine factors associated with a positive screen for post miscarriage depression at Aga Khan University Hospital, Nairobi (AKUH,N).

### Study design

The study was a cross-sectional study looking at the factors that influence occurrence of a positive depression screen after a miscarriage.

### Study setting and participants

The study was conducted at the Aga Khan University hospital Nairobi. The study ran from November 2015 to September 2016. Participants were recruited at the 2 weeks clinic review after a miscarriage at the Gynecology Clinics.

### Inclusion and exclusion criteria

We included women who had a miscarriage (whether pregnancy was terminated due to medical reasons or patients’ choice). We excluded women with other forms of early pregnancy loss and who had previously diagnosed depression.

### Sample size and sampling method

The sample size used was for the prevalence study [8] which the present study forms the second part. We estimated the prevalence of post miscarriage depression occurs in 10–20% of women from published literature [13] . However, A previous study at AKUH,N in postnatal women revealed a depression prevalence of 13% using the Edinburgh Postnatal Depression scale (EPDS) [14]. We assumed that the prevalence of post miscarriage depression may be similar since it was the same setup and used this rate in our sample size calculation.

Sample size was calculated from a formula for estimating a population prevalence [15].

$$ n=\frac{Z^2P\left(1-P\right)}{d^2} $$

Where: n = Required sample size. Z = Z statistic for a 95% confidence interval [1.96). P = Expected prevalence of Post miscarriage depression at AKUH, N. d = Precision around expected prevalence ±0.05. Substituting for the equation:

$$ n=\frac{1.96^2\times 0.13\left(1--0.13\right)}{0.05^2}n=173. $$

The patients were selected by a consecutive sampling method.

### Study procedures

Consent was obtained from participants in the triage room after triage had been done and two tools were used to collect data – a demographics tool and the EPDS. These were administered by the principal investigator and a research assistant. A total of 202 women were approached to derive the sample size of 182–20 women declined to participate in the study.

### Study tools

A demographics tool and the EPDS were used to collect data from the patients.

The demographics tool collected the patients file number, age and other associated factors that may impact on the occurrence of post-miscarriage depression. Depression symptoms were measured using the EPDS [16].

### Data management and analysis

Patient demographics were compared to determine any association between the patients who screened positive for depression to the ones who screened negative. This analysis aimed to determine whether there is an association between the occurrence of depression with the marital status, planning of the pregnancy, social support, mode of treatment of the miscarriage, number of prior miscarriages, mode of conception and prior pregnancy outcome. Univariate analysis was done to compare these clinical variables between the screen - positive and screen - negative women in order to delineate the potential pattern of association among the study subjects. The fisher’s exact test or a chi squared test was used to test the relationship between these two categorical variables depending on the data pattern. Multivariate analysis was done for all potential risk factors in a logistic regression using being screen - positive or screen – negative for depression as the dependent variable. *P* value was set at *P* < 0.05. Data analysis was done using the statistical package for the social sciences (SPSS) version 22. We represented data in graphs and tables.

### Ethical considerations

Ethical approval for this study was obtained from the Ethics committee at the AKUH, N. Participants had consent taken then were recruited into the study. Quality of care received was not influenced by participation or failure to participate in the study. Privacy and confidentiality was maintained using alternative study numbers. Locked cabinets were used to store documents from the study. A referral was done for all screen positive participants to the psychiatrist for further assessment, although this was at the patients’ cost.

## Results

A total of 182 patients were recruited for the study. This represented 90.5% of the total patients with miscarriages approached (201). The patients were recruited from the outpatient gynecological clinics at the AKUH. The prevalence for positive depression screen in post miscarriage patients from the present study is estimated at 34.1% as reported in the other paper in this series [8].

### Analysis of factors associated with post-miscarriage depression

#### Univariate analysis

Univariate analysis was done to determine whether any of the clinical variables was associated with a positive depression screen. This analysis is shown in Table 1. The factors that independently affected depression screen without considering confounder were education level (*p* = 0.039) and mode of conception (*p* = 0.005).

**Table 1:** Univariate analysis of clinical variables and association with positive depression screen

Clinical variable	Odds Ratio	Significance (*p* < 0.05)
Education Level	3.339	0.039
Mode of Conception	8.365	0.005
Age	1.067	0.391
Gestation	1.534	0.094
Marital Status	2.547	0.113
Pregnancy Planning	0.907	0.343
Social Support	0.659	0.419
Others Aware	0.189	0.664
Mode of Treatment	0.308	0.907
Any Prior Miscarriage	1.474	0.225
Immediate Prior Pregnancy Outcome	0.333	0.802

#### Multivariate analysis

Multivariate analysis was performed for all the clinical variables while using depression screen as the dependent variable using logistic regression (Table 2). This was to determine the impact of each of the clinical variables on the depression screen while taking into account the other variables. Using this analysis several of the clinical variables impacted on the depression screen including: age (*p* = 0.09), education level (*p* = 0.01), gestation at miscarriage (*p* = 0.04), marital status (*p* = 0.043), prior miscarriage (*p* = 0.011) and mode of conception (*p* = 0.03).

**Table 2:** Multivariate analysis of clinical variables and association with depression screen

Variable	Description(n)	Prevalence of positive depression screen.	Odds ratio (95% C.I.)	*P* value
Age	Increasing Age	N/A	45.97 (8.2–65.6)	0.009
Gestational age at miscarriage in weeks	Increasing gestational age at miscarriage.	N/A	36.28 (11.6–44.1)	0.004
Level of education	Primary (4)	100%	13.28 (8.7–16.3)	0.001
	Secondary (19	15.8%	0.32 (0.2–1.7)	
	College/University (159)	65.4%	1	
Marital status	Single (34)	36.5%	4.09 (2.3–6.9)	0.043
	Married (148)	23.5%	1	
Pregnancy planning	Planned (132)	37.1%	1	0.205
	Unplanned (50)	26.0%	1.61(− 0.7–1.9)	
Social Support	Lives alone (19)	31.6%	0.34 (−0.1–1.3)	0.561
	Lives with others (163)	34.4%	1	
Others aware of pregnancy	Yes (158)	34.8%	1	0.407
	No (24)	29.2%	0.69 (−0.5–1.1)	
Mode of treatment of Miscarriage	Expectant (40)	35.0%	1	0.753
	Medical (86)	33.7%	2.45(−0.6–2.7)	
	Surgical (45)	31.1%	2.33(−0.6–2.6)	
	Expectant plus Medical (2)	50.0%	2.66(−0.4–2.8)	
	Expectant plus Surgical (5)	40.0%	2.49(−0.5–2.6)	
	Medical plus Surgical (4)	50.0%	2.65(−0.4–2.8)	
Prior Miscarriage	None (124)	31.5%	1	0.011
	1 (44)	43.2%	11.11 (4.6–14.9)	
	2 (6)	16.7%	1.82 (1.4–3.0)	
	3 (8)	37.5%	5.44 (1.9–7.3)	
Mode of Conception	Spontaneous (173)	31.8%	1	0.003
	Assisted (9)	77.8%	9.08 (4.3–11.6)	
Prior Pregnancy Outcome	None (76)	30.3%	1	0.923
	Miscarriage (38)	34.2%	0.48(−0.2–1.3)	
	Live Birth (68)	38.2%	0.41(−0.2–1.2)	

## Discussion

Miscarriages being common in early pregnancy there is growing concern about their mental health implications and hence a budding area of research in obstetrics. The present study was the second part of a study that looked at the prevalence of positive depression screen after a miscarriage [8], and set out to look at the factors that influence this occurrence.

This study looked at which factors may independently impact on the positive screen for depression after a miscarriage. This analysis revealed that only 2 factors could be independent variables to this. They are education level (*p* = 0.039) and mode of conception (*p* = 0.005). Mode of conception has been shown to be a factor influencing occurrence of depression after a miscarriage [6]. Women who have had difficulty in conceiving and hence had assisted conception have been shown to have a predisposition towards developing depression after a miscarriage [6]. This has been majorly attributed to the fact that women who have assisted conception and experience a miscarriage, tend to have feelings of grief, profound aloneness and are concerned on whether they will conceive again [13]. The present study found that education level was associated with occurrence of positive depression screen after a miscarriage meaning the higher the education level the less the chance of a positive depression screen. No studies have looked at the relationship between education level and depression after a miscarriage. However, we can posit that the reason for this may be that if a woman is more educated, she is better placed to understand the cause and clinical outcomes after a miscarriage and hence cope with a miscarriage better than one who is less educated. The other possible reason is that the societal role of a woman changes with education. In that, a woman may be geared towards professional ambitions as opposed to her major role as a parent as she gets more educated. This in turn may lead to a less adverse depressive reaction to a miscarriage. These may explain the lower prevalence among more educated women. The other factors did not seem to independently affect the depression screen after a miscarriage. A few of these factors have been shown to affect positive depression screen after a miscarriage including a younger age at miscarriage [6, 10]. Being single at the time of miscarriage has also been associated with a higher risk of depression but this wasn’t the case in the present study [11]. Finally poor social support has also been associated with depression after a miscarriage [11] but the present study did not delineate that association. These factors ought to have had an impact on depression after a miscarriage. However, their lack of independent impact on depression in the present study can be explained by the fact that the study wasn’t designed and powered to examine these associations therefore the lack of relationship may be an apparent one rather than a true one.

Multivariate analysis was done to delineate potential relationships between the dependent variable of depression screen and the independent variables while taking into other variables as confounders. This was done through logistic regression analysis which revealed that more variables impacted on depression screen in multivariate analysis that did not in Univariate analysis. These included age (*p* = 0.09), education level (*p* = 0.01), gestation at miscarriage (*p* = 0.04), marital status (*p* = 0.043), prior miscarriage (*p* = 0.011) and mode of conception (*p* = 0.03). Some of these factors have been previously associated as risk factors for developing depression after a miscarriage. Age of the woman at miscarriage seemed to impact on the depression screen. This finding has been reported before in multiple studies [6, 11] with a younger age at miscarriage being shown to increase the likelihood of developing depression afterwards. This has been linked to associated social factors such as a younger woman is more likely to be single, live alone and be less educated and hence the miscarriage may a have a heavier bearing with regards to psychiatric morbidity as opposed to an older woman [17]. More so, young age has also been shown to be a risk factor for depressive illness in the general population [18]. Education level has not been studied as a risk factor for depressive illness after a miscarriage but as we discussed earlier, this may impact on the woman’s understanding on the cause and subsequent implications of a miscarriage and hence influence the psychological reaction to it. Gestational age at miscarriage was found to impact on the occurrence of a positive depression screen, in that the older the pregnancy the more the likelihood of depressive symptoms. This has previously been shown in other studies with a gestational age of more than 8 weeks being shown to predispose one to depression as opposed to a gestational age before 8 weeks [19]. This has been attributed to factors such as the couple having formed less attachment to the pregnancy before 8 weeks and there is also less likelihood of undergoing additional treatment procedures such as dilatation and curettage prior to this gestational age which may impact on their psychological reaction after the loss [20, 21]. Marital status seemed to impact on the occurrence of positive depression screen after a miscarriage in the present study. This has been observed before with being single at the time of a miscarriage impacting on the occurrence of depression afterwards [11, 17]. This was further attributed to the social support one has a after a miscarriage which may impact on the occurrence of depression as discussed previously. The presence of a prior miscarriage also seemed to be a factor determining the occurrence of positive depression screen in our study. This was shown previously with women who had a previous miscarriage being shown to be predisposed to depression after a miscarriage [7]. This was further shown to increase with the number of prior miscarriages and was attributed to the growing anxiety and concern about having a miscarriage in a current pregnancy from prior experience [7]. Mode of conception was also shown to impact on occurrence of positive depression screen after a miscarriage in the multivariate analysis. This was also true in the Univariate analysis, a factor explained earlier, as the impact of mode of conception on the reaction after a miscarriage [13]. The other factors that still didn’t seem to impact on the occurrence of a miscarriage were pregnancy planning, social support, others being aware of the pregnancy and prior pregnancy outcome and their prior impact of lack of had been discussed earlier. Although these trends may seem apparent in the present study, it is important to note that this study wasn’t powered to investigate them but may point out on potential associations that may warrant further investigation in our population. However, it is reassuring to note that the factors identified to be associated with a positive depression screen in our study are similar to those identified in other studies.

### Limitations and future directions

The present study was limited in terms of sample size to conclusively look at these factors independently to determine the one with the biggest impact on positive depression screen. However, this may be the basis of a future study to evaluate these factors in greater detail.

## Conclusion

In conclusion, factors that seem to impact on the positive depression screen include a younger age, low education level, an older gestational age at miscarriage, being single, an assisted mode of conception and prior miscarriage. These factors may be used to triage women after a miscarriage in order to pick up those who may screen positive for depression after a miscarriage which is important in a clinical set up.

## Abbreviations

AKUH,N: Aga Khan University Hospital, Nairobi
EPDS: Edinburgh Postnatal Depression scale
SPSS: Statistical Package for the Social Sciences

## References

[1] Tong S, Kaur A, Walker SP, Bryant V, Onwude JL, Permezel M (2008). Miscarriage risk for asymptomatic women after a normal first-trimester prenatal visit. Obstet Gynecol.

[2] Zinaman MJ, Clegg ED, Brown CC, O'Connor J, Selevan SG (1996). Estimates of human fertility and pregnancy loss. Fertil Steril.

[3] Coleman PK (2011). Abortion and mental health: quantitative synthesis and analysis of research published 1995-2009. The British journal of psychiatry : the journal of mental science.

[4] Bianchi-Demicheli F (2007). Psychiatric and psychological consequences of abortion. Revue medicale suisse.

[5] McCarthy FP, Moss-Morris R, Khashan AS, North RA, Baker PN, Dekker G (2015). Previous pregnancy loss has an adverse impact on distress and behaviour in subsequent pregnancy. BJOG : an international journal of obstetrics and gynaecology.

[6] Sham A, Yiu M, Ho W (2010). Psychiatric morbidity following miscarriage in Hong Kong. Gen Hosp Psychiatry.

[7] Neugebauer R (2003). Depressive symptoms at two months after miscarriage: interpreting study findings from an epidemiological versus clinical perspective. Depression and anxiety.

[8] Mutiso SK, Murage A, Mukaindo AM (2018). Prevalence of positive depression screen among post miscarriage women- a cross sectional study. BMC psychiatry.

[9] Reardon DC, Cougle JR, Rue VM, Shuping MW, Coleman PK, Ney PG (2003). Psychiatric admissions of low-income women following abortion and childbirth. CMAJ : Canadian Medical Association journal = journal de l'Association medicale canadienne.

[10] Pedersen W (2008). Abortion and depression: a population-based longitudinal study of young women. Scandinavian journal of public health.

[11] Reardon DC, Cougle JR (2002). Depression and unintended pregnancy in the National Longitudinal Survey of youth: a cohort study. BMJ (Clinical research ed).

[12] Rees DI, Sabia JJ (2007). The relationship between abortion and depression: new evidence from the fragile families and child wellbeing study. Med Sci Monit.

[13] Freda MC, Devine KS, Semelsberger C (2003). The lived experience of miscarriage after infertility. MCN The American journal of maternal child nursing.

[14] Khadija W. Prevalence of Postpartum Depression using the Edinburgh Postpartum Depression scale at the Aga Khan University Hospital, Nairobi Aga Khan University Hospital; 2011.

[15] Pourhoseingholi MA, Vahedi M, Rahimzadeh M. Sample size calculation in medical studies. Gastroenterol Hepatol From Bed To Bench. 2013;6(1):14–7. PMC4017493 24834239

[16] Cox JL, Holden JM, Sagovsky R (1987). Detection of postnatal depression. Development of the 10-item Edinburgh postnatal depression scale. The British journal of psychiatry : the journal of mental science..

[17] Rich-Edwards JW, Kleinman K, Abrams A, Harlow BL, McLaughlin TJ, Joffe H (2006). Sociodemographic predictors of antenatal and postpartum depressive symptoms among women in a medical group practice. J Epidemiol Community Health.

[18] Brown DR, Ahmed F, Gary LE, Milburn NG (1995). Major depression in a community sample of African Americans. Am J Psychiatry.

[19] Huffman CS, Schwartz TA, Swanson KM (2015). Couples and Miscarriage: the influence of gender and reproductive factors on the impact of miscarriage. Women's health issues : official publication of the Jacobs Institute of Women's Health.

[20] Gerber-Epstein P, Leichtentritt RD, Benyamini Y (2008). The experience of miscarriage in first pregnancy: the Women's voices. Death Studies.

[21] Smith LF, Frost J, Levitas R, Bradley H, Garcia J (2006). Women's experiences of three early miscarriage management options: a qualitative study. The British journal of general practice : the journal of the Royal College of General Practitioners.

